# Bone Marrow Alterations and Lower Endothelial Progenitor Cell Numbers in Critical Limb Ischemia Patients

**DOI:** 10.1371/journal.pone.0055592

**Published:** 2013-01-31

**Authors:** Martin Teraa, Ralf W. Sprengers, Peter E. Westerweel, Hendrik Gremmels, Marie-José T. H. Goumans, Tom Teerlink, Frans L. Moll, Marianne C. Verhaar

**Affiliations:** 1 Department of Nephrology & Hypertension, University Medical Center Utrecht, Utrecht, The Netherlands; 2 Department of Vascular Surgery, University Medical Center Utrecht, Utrecht, The Netherlands; 3 Department of Radiology, University Medical Center Utrecht, Utrecht, The Netherlands; 4 Department of Haematology, University Medical Center Utrecht, Utrecht, The Netherlands; 5 Department of Molecular Cell Biology, Leiden University Medical Center, Leiden, The Netherlands; 6 Department of Clinical Chemistry, VU University Medical Center, Amsterdam, The Netherlands; Centro Cardiologico Monzino, Italy

## Abstract

**Background:**

Critical limb ischemia (CLI) is characterized by lower extremity artery obstruction and a largely unexplained impaired ischemic neovascularization response. Bone marrow (BM) derived endothelial progenitor cells (EPC) contribute to neovascularization. We hypothesize that reduced levels and function of circulating progenitor cells and alterations in the BM contribute to impaired neovascularization in CLI.

**Methods:**

Levels of primitive (CD34^+^ and CD133^+^) progenitors and CD34^+^KDR^+^ EPC were analyzed using flow cytometry in blood and BM from 101 CLI patients in the JUVENTAS-trial (NCT00371371) and healthy controls. Blood levels of markers for endothelial injury (sE-selectin, sICAM-1, sVCAM-1, and thrombomodulin), and progenitor cell mobilizing and inflammatory factors were assessed by conventional and multiplex ELISA. BM levels and activity of the EPC mobilizing protease MMP-9 were assessed by ELISA and zymography. Circulating angiogenic cells (CAC) were cultured and their paracrine function was assessed.

**Results:**

Endothelial injury markers were higher in CLI (P<0.01). CLI patients had higher levels of VEGF, SDF-1α, SCF, G-CSF (P<0.05) and of IL-6, IL-8 and IP-10 (P<0.05). Circulating EPC and BM CD34^+^ cells (P<0.05), lymphocytic expression of CXCR4 and CD26 in BM (P<0.05), and BM levels and activity of MMP-9 (P<0.01) were lower in CLI. Multivariate regression analysis showed an inverse association between IL-6 and BM CD34^+^ cell levels (P = 0.007). CAC from CLI patients had reduced paracrine function (P<0.0001).

**Conclusion:**

CLI patients have reduced levels of circulating EPC, despite profound endothelial injury and an EPC mobilizing response. Moreover, CLI patients have lower BM CD34^+^-cell levels, which were inversely associated with the inflammatory marker IL-6, and lower BM MMP-9 levels and activity. The results of this study suggest that inflammation-induced BM exhaustion and a disturbed progenitor cell mobilization response due to reduced levels and activity of MMP-9 in the BM and alterations in the SDF-1α/CXCR4 interaction contribute to the attenuated neovascularization in CLI patients.

## Introduction

Critical limb ischemia (CLI) is a major health care problem, associated with a high risk of limb loss [1] as well as a high short-term cardiovascular ischemic event rate and increased mortality [2]–[4]. CLI is caused by obstruction of lower extremity arteries – most often due to atherosclerosis – in combination with a yet largely unexplained impaired ischemic neovascularization response. Postnatal neovascularization in response to tissue ischemia occurs not only by migration and proliferation of resident mature endothelial cells but also involves bone marrow (BM) derived endothelial progenitor cells (EPC) [5]. In response to hypoxia, the local production of chemokines and growth factors such as stromal cell-derived factor-1α (SDF-1α) and vascular endothelial growth factor (VEGF) is upregulated, leading to elevated blood levels. In the BM microenvironment this induces release and activation of matrix metalloproteinases (MMPs) causing EPC, which are positive for the SDF-1α receptor CXCR4 and VEGF receptor 2 (VEGFR-2, KDR) to mobilize to the circulation [6]. EPC subsequently contribute to neovascularization, either by physical incorporation into the endothelial layer or by excretion of paracrine factors that stimulate proliferation of resident endothelial cells [5], the latter being likely the paramount mechanism [7], [8], occurring in delicate concert with other circulating cells, such as monocytes [9].

Patients with CLI have a large burden of cardiovascular risk factors and endothelial dysfunction, characterized by reduced nitric oxide (NO) bioavailability. The presence of cardiovascular risk factors and overt cardiovascular disease have been associated with reduced numbers and impaired function of circulating EPC [10]–[14]. Although it has been clearly demonstrated that circulating EPC increase in response to acute tissue injury or ischemia [15]–[17], studies that have reported on EPC number and function in patients with chronic continuous ischemia as a result of ongoing cardiovascular disease, as is the case in chronic CLI, are scarce. In patients with chronic ischemic heart disease, the number of circulating EPC was reduced [18], [19]. Thus far, only few small studies have reported reduced numbers of circulating EPC in chronic CLI [12], [13], [20], [21]. Only Fadini et al. reported on circulating angiogenic cells (CAC), which like circulating EPC exert their angiogenic effects mainly via a paracrine mechanism [22], and found reduced clonogenic and adhesive function of these cells in 15 patients with PAD, however the proportion of CLI patients was not defined, as compared to control subjects [13]. Levels of progenitor cells in the BM of patients with cardiovascular disease have rarely been studied relative to the healthy situation. Heeschen et al. observed no differences in the percentage of BM-MNC expressing CD34 in 18 patients with ischemic cardiomyopathy compared to healthy controls, but significant impairment of BM progenitor cell function [19]. This observation was later confirmed by Kissel and colleagues in a population of 94 ischemic cardiomyopathy patients [18]. Oda et al. recently reported no significant differences in the fraction of BM-MNC expressing CD34, but hypocellularity of the BM and hence an absolute reduction of BM CD34^+^-cell content in a small heterogeneous group of 16 CLI patients (atherosclerosis obliterans and other causes of CLI) compared to healthy controls [23].

We hypothesized that in patients with CLI circulating EPC are dysfunctional and numerically reduced due to a direct negative impact of this systemic disease on the BM, causing BM exhaustion, impaired progenitor cell mobilization, and cellular dysfunction. In 101 CLI patients participating in an ongoing trial [24] on the effects of BM mononuclear cell (MNC) administration, we investigated the numbers of hematopoietic and endothelial progenitor cells in BM and peripheral blood (PB), as well as CAC outgrowth from PB-MNC and their paracrine function, in comparison to healthy controls. EPC mobilizing (eg SDF-1α and VEGF) and inflammatory (eg interleukin-6 and interleukin-8) factors were assessed in PB and MMPs (MMP-2 and 9) were assessed in BM as factors mediating and CXCR4 and CD26 expression as factors modulating [25], [26] EPC mobilization from the BM.

## Methods

### Study subjects

We collected PB and BM samples from 101 patients with CLI participating in the JUVENTAS study; a trial evaluating the clinical effects of intra-arterial infusion of BM-MNC in CLI (clinicaltrials.gov NCT00371371) [24]. Patients with chronic CLI, an ankle-brachial index (ABI) of 0.6 or less, or an unreliable index (non-compressible or not in proportion to the Fontaine classification), and who were not candidate for conventional revascularization are included in this trial. Exclusion criteria were a history of neoplasm or malignancy in the past 10 years, concomitant disease with life expectancy of less than one year, inability to obtain sufficient BM aspirate, known infection with human immunodeficiency virus, hepatitis B or C virus, and an impossibility to complete follow-up.

Control PB samples were collected from 37 gender- and age-matched healthy subjects. Control BM samples were obtained from patients without a history of peripheral arterial disease undergoing orthopaedic surgery (n = 12 and n = 8 for flow cytometry and ELISA, respectively).

In patients with CLI a total volume of 100 ml BM was aspirated from the iliac crest under local anaesthesia and conscious sedation for use in the clinical trial. Seven ml of BM was reserved for (functional) characterization of BM cells. In the BM control group approximately 15 ml of BM was harvested from the surgical site under general anaesthesia.

CLI patients and PB controls were asked to fill out health-related questionnaires, and underwent a brief physical examination and laboratory testing (complete blood count, liver enzymes, kidney function, lipid spectrum, and glucose and homocysteine level). BM control subjects filled out a similar questionnaire and underwent physical examination and laboratory testing as part of the hospital's pre-operative screening program.

### Ethics statement

The study has been approved by the Medical Ethics Committee of the University Medical Center Utrecht, was performed conform the Declaration of Helsinki, and all patients gave written informed consent.

### Plasma and serum measurements

Biochemical parameters (liver enzymes, kidney function, lipid spectrum, and glucose and homocysteine level) were measured using standard clinical laboratory procedures.

All PB samples were collected in potassium-EDTA or serum tubes. All BM samples were collected in a sodium heparin solution. Markers for vascular endothelial activation or injury, soluble E-Selectin (sE-selectin), soluble intercellular adhesion molecule-1 (sICAM-1), soluble vascular cell adhesion molecule-1 (sVCAM-1) (R&D Systems, Minneapolis, MN, USA) and thrombomodulin (Diaclone, Stamford, CT, USA), were determined in PB samples using commercially available enzyme immunoassay (ELISA) kits according to manufacturer's instructions. Additionally, PB SDF-1α levels, total (active and inactive) BM plasma MMP-2 (gelatinase A) and 9 (gelatinase B) levels were measured using commercially available ELISA kits (R&D Systems) according to manufacturer's instructions.

Customized group I and II bio-plex multiplex cytokine assays (Bio-rad Labarotories, Hercules, CA, USA) were used to measure levels of cytokines and growth factors (Basic fibroblastic growth factor [FGF-b], Granulocyte-colony stimulating factor [G-CSF], Growth regulated oncogene-alpha [GRO-α], Hepatocyte growth factor [HGF], Interleukin-6 [IL-6], Interleukin-8 [IL-8], Interferon gamma-induced protein 10 [IP-10], Monocyte chemotactic protein 1 [MCP-1], Platelet-derived growth factor-bb [PDGF-bb], Regulated upon activation normal T-cell expressed, and presumably secreted [RANTES], Stem cell factor [SCF], Stem cell growth factor-beta [SCGF-β], Tumor necrosis factor-alpha [TNF-α], Tumor necrosis factor related apoptosis inducing ligand [TRAIL], and Vascular endothelial growth factor [VEGF]) in PB serum, according to manufacturer's instructions.

Asymmetric dimethylarginine (ADMA) competitively inhibits production of NO from the substrate arginine by NO synthase, whereas symmetric dimethylarginine (SDMA) may limit NO production by competing with arginine for cellular uptake. Arginine, ADMA, and SDMA were determined in PB plasma using high-performance liquid chromatography (HPLC) with fluorescence detection as previously described [27], using modified chromatographic separation conditions [28].

### Zymographic analysis

Gelatinolytic activities of BM plasma samples were assessed by zymography as described previously (Fig. S1) [29]. In short, BM plasma samples were diluted 10 times with phosphate buffered saline and supplemented with loading buffer (0.25 mol/L Tris-HCL, 8% SDS, 40% glycerol, and 0.004% Bromophenol Blue). PageRuler pre-stained protein ladder #SM0671 (Fermentas Life Sciences, Burlington, Ontario, Canada) was used to determine the weights of the gelatinases. Loading buffer was used as negative control and 5 µl of 1∶25000 83 kDa active MMP-9 (Calbiochem, La Jolla, CA, USA) was used as positive control. Proteins were separated on a 4% polyacramide stacking gel onto a 10% polyacramide running gel, copolymerized with 2 mg/mL gelatine (Sigma-Aldrich, St. Louis, MO, USA). Subsequently gels were incubated overnight at 37°C in Brij solution (10 mmol/L CaCl_2_, 0.05% Brij 35 solution [Sigma-Aldrich], 50 mmol/L Tris-HCL pH 7.4) and stained (25% MeOH, 15% AcOH, and 1% Coomassie Blue). Gels were photographed and picture analysis was performed using NIH ImageJ 1.42q software. The lytic zones of the BM samples were normalized to the positive control and expressed as arbitrary units (AU).

### Flow cytometry analysis of circulating and bone marrow progenitor cells

A volume of 100 µl of PB or a BM volume containing 1×10^6^ white blood cells was incubated with anti-CD34-FITC (BD Pharmingen, San Diego, CA, USA), anti-KDR-PE (R&D Systems) and anti-CD45-PE-Cy7 (BD Pharmingen) antibodies for 45 minutes. Erythrocytes were lysed in an ammonium chloride buffer and remaining cells were washed and analyzed by flow cytometry (FC 500, Beckman Coulter, Fullerton, CA, USA). Circulating EPC were identified as CD34^+^KDR^+^ cells in the lymphocytic region of the forward/sideward scatter plot (Fig. S2) [30]. To assess primitive hematopoietic progenitors, 100 µl of PB or a BM volume containing 1×10^6^ white blood cells was incubated with anti-CD133-PE (Miltenyi Biotec, Bergisch Gladbach, Germany). Primitive hematopoietic progenitors were identified as CD133^+^ cells in the lymphocytic region of the forward/sideward scatter plot. Cell numbers were quantified per millilitre of blood and relative to 1×10^6^ granulocytic events in the BM. Granulocytic events were identified based on their typical distribution on the forward/sideward scatter plot. Measurements were performed in duplo and results were averaged. Isotype-stained samples served as negative controls.

Expression of CXCR4 and CD26 was assessed by incubating 100 µl of EDTA blood or a BM volume containing 1×10^6^ white blood cells with anti-CXCR4-PE (BD Pharmingen) and anti-CD26-FITC (AbD Serotec, Oxford, United Kingdom). Expression of CXCR4 and CD26 was assessed in the complete MNC population as well as in the lymphocyte and monocyte subpopulations. Lymphocytes were identified according to their distribution on the forward/sideward scatter plot. Cells were also incubated with anti-CD14-ECD (Immunotech, Coulter, France) to allow for the separation of monocytes.

### Circulating angiogenic cell quantification and functional characterization

CAC numbers obtained from PB-MNC were assessed as described previously [31]. In brief, MNC were isolated from PB samples using density gradient centrifugation (Histopaque 1077, Sigma-Aldrich). To evaluate CAC numbers in culture, MNC were seeded on a human fibronectin (Sigma-Aldrich) coated 6-well plate (10×10^6^ per well), using EGM-2 medium (Cambrex, Walkersville, MD, USA), supplemented with accompanying aliquots, 20% fetal bovine serum (Invitrogen, Carlsbad, CA, USA), 100 ng/ml recombinant VEGF-165 (R&D Systems) and antibiotics. Medium was changed after 4 days to remove non-adherent cells. After 7 days, medium was removed and the CAC were placed on serum free medium (EBM-2 with hEGF, hydrocortisone, GA-1000, R3-IGF-1, ascorbic acid, heparin and antibiotics) overnight. Finally, this conditioned medium (CM) was collected and stored for functional experiments. In the applied protocol essentially every adherent cell after the 7-day culture period stained double-positive for acetylated-LDL (ac-LDL) and Ulex europaeus lectin (UEA-lectin; Fig. S3). For the experiments in this study CAC obtained using this protocol were detached using trypsin and cell scraping, and automatically counted on a hemocytometer (Cell-dyn 1800, Abbott laboratories, Abbott Park, IL, USA).

To examine the capacity of CAC to excrete paracrine factors that stimulate endothelial cell migration, an in vitro scratch wound assay was performed as described previously [32]. Human microvascular endothelial cells (HMEC, Centers for Disease Control and Prevention, Atlanta, GA, USA) were grown to confluence on fibronectin coated 48-well plates, using MCDB-131 medium, supplemented with 10% fetal bovine serum, 5% L-glutamine, 0.1% hEGF, 0.1% hydrocortisone, and antibiotics. Lines were drawn on the bottom of each well to serve as a reference during image acquisition. A straight mechanical scratch was created using a p200 pipette tip and the HMEC monolayer was carefully washed with phosphate buffered saline. CM obtained from the CAC cultures was subsequently placed on the cells. The scratched area was photographed using a light microscope at start and after 6 hours of incubation at 37°C. CAC culture medium and CAC serum free culture medium served as positive and negative controls, respectively. After 6 hours the extent of scratch closure was determined relative to the scratch width before incubation, using Image-Pro Plus software version 3.0 (Media Cybernetics, Bethesda, MD, USA). Each sample was measured in two separate wells and each scratch was examined at two separate reference points per well. Cell closure measurements were averaged for data analysis.

### Statistical analysis

Continuous data are expressed as mean ± standard deviations (SD) or median and 25^th^ and 75^th^ percentiles (P25–P75), depending on the normality of the data. Continuous variables were tested whether they fulfilled the assumptions for parametric tests. A Levene's test was used to test for equality of variances between the groups. Independent samples *t*-tests were used to test for differences of parametric variables between two groups. The Mann-Whitney *U*-test was used to test for differences of non-parametric variables between groups. Categorical variables were compared using a chi-square test. False discovery rate (FDR) control of the comparisons of chemokines, growth factors and progenitor cell numbers between CLI patients and healthy controls was performed using the Benjamini and Hochberg method.

Univariate analyses were performed by calculating Spearman's rho, since the majority of variables did not meet the assumptions for the Pearson correlation. Multivariate analyses were performed by performing step forward multivariate linear regression analyses. Variables that did not meet the assumption of normality and constant variance of the residuals were Log_e_-transformed prior to multivariate linear regression. P-values<0.05 were considered statistically significant. All analyses were performed using SPSS for Windows version 15.0 (SPSS Inc., Chicago, IL, USA).

## Results

### Patient characteristics

Patient characteristics are shown in Table 1. Age and sex did not differ between CLI patients and healthy controls. Besides total and LDL-cholesterol levels, which were higher in the healthy controls, classical cardiovascular risk factors, such as presence of hyperhomocysteinemia, hypercholesterolemia and diabetes were all significantly more prominent in CLI patients.

**Table 1:** Subject characteristics.

	CLI patients (n = 101)	Healthy controls (n = 37)	BM controls (n = 12)
Age (years)	65.3±11.8	62.4±14.4	58.6±13.8
Male gender	71 (70%)	23 (62%)	7 (58%)
History of cardiovascular disease			
*Previous revascularisation of affected leg*	85 (85%)	0 (0%)*	0 (0%)*
*CABG*	16 (16%)	0 (0%)*	1 (8%)
*Congestive heart failure*	6 (6%)	0 (0%)	0 (0%)
*Myocardial infarction or angina pectoris*	35 (35%)	0 (0%)*	3 (25%)
*TIA*	6 (6%)	0 (0%)	0 (0%)
*Stroke*	8 (8%)	0 (0%)	0 (0%)
*End stage renal disease*	3 (3%)	0 (0%)	0 (0%)
Body mass index (kg/m^2^)	26.6±4.7	24.0±2.9*	25.4±2.4
Currently smoking	26 (26%)	0 (0%)*	3 (25%)
Diabetes	40 (40%)	0 (0%)*	2 (17%)
Hypertension	89 (88%)	2 (5%)*	7 (58%)*
Systolic blood pressure (mmHg)	138±22	129±22*	137±14
Hypercholesterolemia	95 (94%)	2 (5%)*	†
Total cholesterol (mmol/l)	4.3±1.1	5.0±1.0*	†
HDL-cholesterol (mmol/l)	1.1 (0.9–1.5)	1.4 (1.1–1.6)*	†
LDL-cholesterol (mmol/l)	2.3±0.9	3.3±0.8*	†
Triglycerides (mmol/l)	1.3 (0.9–1.9)	0.6 (0.6–0.9)*	†
Statins	90 (89%)	0 (0%)*	3 (25%)*
ACE inhibitors/Angiotensin receptor blocker	55 (55%)	0 (0%)*	4 (33%)
Beta-blockers	46 (46%)	0 (0%)*	4 (33%)
Diuretics	49 (49%)	0 (0%)*	1 (8%)*
Anticoagulants	36 (36%)	0 (0%)*	1 (8%)
Antiplatelet aggregation therapy	71 (70%)	0 (0%)*	2 (17%)*
Homocysteine (µmol/l)	14.9 (12.1–19.9)	12.1 (9.6–13.9)*	†
*Currently hyperhomocysteinemic*	33 (33%)	0 (0%)*	†
Creatinine (µmol/l)	90.5 (76.0–117.0)	81 (76.0–91.5)*	88 (74.0–95.0)
Hemoglobin (mmol/l)	8.1±1.1	8.9±0.8*	7.5±1.1
Fontaine classification (grade III/IV)	38/63	0/0*	0/0*
Rutherford classification (grade 4/5/6)	38/60/3	0/0/0*	0/0/0*
Ankle-brachial pressure index	0.47±0.26	†	†

Values are presented as absolute numbers and percentage (n [%]) for categorical variables and mean ± SD or medians and P25–P75, unless otherwise specified.

Presence of hypertension, hypercholesterolemia, and hyperhomocysteinemia were determined at the time of inclusion. Hypertension was defined as having a systolic blood pressure >140 mmHg or taking antihypertensive medication. Hypercholesterolemia was defined as having a total cholesterol level >6.5 mmol/l or taking cholesterol reducing medication. Hyperhomocysteinemia was defined as having a homocysteine level >19 µmol/l for men or >17 µmol/l for women.

* P<0.05 compared to JUVENTAS patients,

† data not available.

#### Serum and plasma markers for endothelial activation

Levels of markers for vascular endothelial activation or injury, sICAM-1, sVCAM-1, sE-selectin and thrombomodulin, were significantly higher in CLI patients compared to healthy controls (Table 2). Levels of sVCAM-1 were significantly higher in CLI patients with ulcers or gangrene (Fontaine grade IV) than in CLI patients with ischemic rest pain only (Fontaine grade III) (607±185 ng/ml vs 481±123 ng/ml; P = 0.005). Diabetic CLI patients had significantly higher sVCAM-1 levels in comparison to non-diabetic CLI patients (647±200 ng/ml vs 491±127 ng/ml; P = 0.006). In addition sVCAM-1 levels correlated with ABI, homocysteine and creatinine levels (P = 0.049, P = 0.003 and P = 0.001, respectively; Table S1).

**Table 2:** Endothelial dysfunction markers, chemokines and MMPs.

	CLI patients	Controls	P-value
**Endothelial markers**			
sE-selectin (ng/ml)	37.3 (27.6–45.4)**	29.2 (21.7–33.7)	0.002
sICAM-1 (ng/ml)	266 (180–295)**	136 (113–163)	<0.0001
sVCAM-1 (ng/ml)	546±169**	395±108	<0.0001
thrombomodulin (ng/ml)	0.42 (0.18–0.58)**	0.15 (0.04–0.36)	0.001
**Chemokines and Growth Factors**			
FGF-b (pg/ml)	26.9 (11.7–37.8)	22.1 (10.8–40.9)	0.736
G-CSF (pg/ml)	21.0±14.7*	15.6±24.0	0.017
GRO-a (pg/ml)	120±84**	67±62	0.001
HGF (pg/ml)	541 (442–812)**	297 (208–392)	<0.0001
IL-6 (pg/ml)	6.6 (1.8–14.5)*	4.1 (1.0–7.0)	0.023
IL-8 (pg/ml)	17.0 (11.2–24.5)**	10.6 (7.9–13.8)	<0.0001
IP-10 (pg/ml)	951 (625–1604)**	688 (480–840)	<0.0001
MCP-1 (pg/ml)	55.2±27.9	48.0±27.4	0.191
PDGF-bb (ng/ml)	11.2 (8.7–15.3)	11.5 (8.9–15.7)	0.845
RANTES (ng/ml)	7.7 (6.1–9.6)	8.1 (6.2–11.3)	0.438
SCF (pg/ml)	150 (115–192)*	124 (91–150)	0.013
SCGF-β (ng/ml)	14.5 (10.4–20.0)	15.2 (11.0–19.3)	0.689
SDF-1α (ng/ml)	2.6 (2.2–3.0)*	2.4 (2.2–2.7)	0.012
TNF-α (pg/ml)	9.9 (1.8–22.0)	12.2 (3.1–22.0)	0.867
TRAIL (pg/ml)	92 (68–126)**	124 (98–150)	0.005
VEGF-A (pg/ml)	150±145*	124±89	0.017
**MMPs**			
MMP-2 (BM) (ng/ml)	129 (117–161)**	95 (78–114)	0.001
MMP-9 (BM) (ng/ml)	381 (270–520)**	660 (570–799)	<0.0001
**MMP activity**			
MMP-9 (BM) (AU)	414±205**	717±194	0.002
Pro-MMP-9 (BM) (AU)	57±43	110±95	0.167
MMP-2 (BM) (AU)	193±74	133±82	0.064

Data represent means ± SD or medians and P25–P75.

* P<0.05 and

** P<0.01 compared to control subjects. Endothelial markers were assessed in a subgroup of 54 CLI-patients and 22 controls.

### Widespread inflammatory and progenitor cell mobilizing response in CLI

CLI patients show markedly increased levels of factors associated with progenitor cell recruitment and mobilization, such as SDF-1α, VEGF, SCF, G-CSF, and HGF (Table 2). Inflammatory cytokines, such as IL-6, Il-8, and IP-10 were significantly higher in CLI patients compared to healthy controls, whereas tumor necrosis factor-related apoptosis-inducing ligand (TRAIL), which has been associated with anti-inflammatory and anti-atherosclerotic effects was lower in CLI [33]. Levels of IL-8 were higher in CLI patients with diabetes compared to non-diabetic patients (18.7 [13.4–25.2] pg/ml vs 14.3 [9.9–22.5] pg/ml, P = 0.048) and IL-6 levels showed a similar trend (10.5 [3.9–15.3] pg/ml vs 4.5 [1.4–11.2] pg/ml, P = 0.050). Additionally, both cytokines showed a trend towards an association with disease severity (rho = 0.310, P = 0.002; rho = 0.188, P = 0.059, for IL-6 and IL-8, respectively).

The progenitor cell mobilizing factors SCF and GRO-α were significantly higher in Fontaine grade IV patients compared to patients with Fontaine grade III (131 [70–173] pg/ml vs 169 [116–212] pg/ml, P = 0.025; 93±105 pg/ml vs 131±103 pg/ml, P = 0.043, respectively; Table S2).

### Reduced L-arginine blood levels in CLI

Arginine was lower in PB plasma of CLI patients than in healthy controls (Table 3). No differences were found in PB for the arginine analogues ADMA and SDMA. Arginine levels were significantly lower in patients with ulcers or gangrene compared to patients with rest pain only (58.1 [46.1–68.6] µmol/l vs 68.5 [61.3–80.6] µmol/l, P = 0.005), while SDMA levels were higher in the Fontaine grade IV patients (0.54 [0.46–0.65] µmol/l vs 0.62 [0.54–0.87] µmol/l, P = 0.026; Table S1).

**Table 3:** Arginine analogues.

	CLI patients	Controls	P-value
Arginine (µmol/l)	62.1 (51.1–74.4)**	78.7 (68.7–86.6)	<0.0001
ADMA (µmol/l)	0.44 (0.41–0.49)	0.46 (0.42–0.51)	0.327
SDMA (µmol/l)	0.59 (0.49–0.76)	0.59 (0.53–0.63)	0.564

Data represent medians and P25–P75.

** P<0.01 compared to control subjects. Arginine analogues were measured in the blood of 74 CLI patients and in 23 healthy controls.

### Decreased EPC levels in CLI and reduced hematopoietic stem cell levels in BM

**Figure 1:**
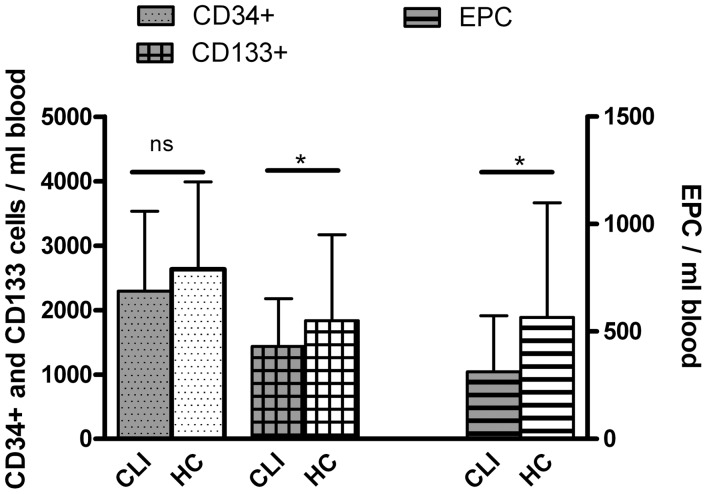
Lower circulating progenitor cell levels in CLI patients. Data represent median and P75. Circulating progenitor cell numbers in CLI patients (n = 101) and healthy controls (HC; n = 37). The number of circulating CD133^+^ and CD34^+^KDR^+^ EPC was significantly reduced in CLI patients (* P<0.05). No significant (ns) differences in circulating numbers were observed for CD34^+^ cells.

Circulating levels of CD34^+^ hematopoietic stem cells (HSC) were not different between CLI patients and healthy controls (Table 4; Fig. 1). However, the proportion co-expressing the endothelial lineage marker KDR was significantly lower in CLI patients, resulting in lower circulating EPC (CD34^+^KDR^+^-double-positive EPC; 313 [124–574] cells/ml vs 566 [198–1099] cells/ml, P = 0.010). In addition the amount of circulating CD133^+^ primitive hematopoietic cells (ie hemangioblasts) was significantly lower in CLI patients. In a step forward multivariate linear regression model including all patient characteristics (Table 1) age, BMI, and use of antiplatelet drugs were negatively correlated with the amount of CD34^+^-cells, no correlations of patient characteristics were observed with circulating EPC levels, and CD133^+^-cells were negatively associated with disease severity (Table S3 shows results of univariate correlations).

**Table 4:** Progenitor cells.

	CLI patients	Controls	P-value
**Peripheral Blood**			
CD34 (cells/ml)	2294 (1630–3530)	2632 (1619–3989)	0.645
%KDR of CD34	12.4 (6.3–27.7)*	22.5 (11.5–36.8)	0.016
CD34KDR (cells/ml)	313 (124–574)*	566 (198–1099)	0.010
CD133 (cells/ml)	1437 (946–2176)*	1836 (1112–3169)	0.040
**Bone Marrow**			
CD34 (cells/1*10∧6gran)	738 (459–997)*	990 (688–1561)	0.018
%KDR of CD34	1.5 (0.7–3.1)	1.1 (0.9–2.8)	0.932
CD34KDR (cells/1*10∧6gran)	10.0 (4.9–21.9)	12.0 (6.5–36.0)	0.349
CD133 (cells/1*10∧6gran)	107 (67–168)	80 (33–210)	0.292

Data represent medians and P25–P75.

* P<0.05 compared to control subjects. Progenitor cells were assessed in 101 CLI patients and in 37 and 12 healthy controls for PB and BM, respectively.

**Figure 2:**
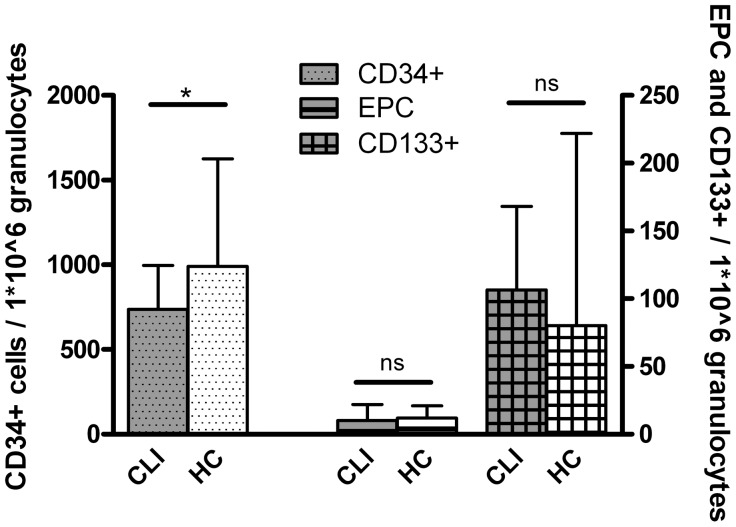
BM CD34^+^ progenitor cell are reduced in CLI patients. Data represent median and P75. BM progenitor cell numbers in CLI patients (n = 101) and healthy controls (HC; n = 12). CD34^+^ cells were significantly reduced (* P<0.05) in CLI patients compared to health controls. No significant (ns) differences in CD133^+^-cells and CD34^+^KDR^+^ EPC in the BM.

The BM of CLI patients contained significantly lower numbers of CD34^+^-cells compared to that of healthy controls (738 [459–997] cells/1×10^6^ granulocytes vs 990 [688–1561] cells/1×10^6^ granulocytes, P = 0.018). No differences for CD34^+^KDR^+^-cells and CD133^+^-cells were observed (Table 4; Fig. 2). Of the patient characteristics there was only a negative association of CD34^+^-cells with ABI and Log_e_ converted creatinine levels. The other cell types in BM were not correlated with any of the demographic data (Table S3).

### Relationship between cell populations and humoral factors

Since MMP-9 and MMP-2 have been suggested to be involved in EPC mobilization [6], [34] levels and activity were studied in the BM. MMP-9 was found to be significantly lower in CLI patients compared to healthy controls, while MMP-2 was significantly higher (Table 2; Table S4 shows univariate correlations of MMP-levels with patient characteristics). Both levels and activity of these MMPs were not related to circulating EPC levels, however a weak positive correlation of MMP-9 with CD34^+^-cells in circulation was observed (rho = 0.255, P = 0.011).

In order to find explanatory factors for the reduction of circulating CD34^+^KDR^+^-cells and BM CD34^+^-cells in CLI patients, correlations with cytokines and growth factors were studied. In univariate analysis there was a significant positive correlation of circulating CD34^+^KDR^+^-cells with SCGF-β, which remained the only significant predictor in a step forward multivariate linear regression model, including all cytokines and growth factors. In CLI patients the levels of BM CD34^+^-cells, which were reduced compared to healthy controls, were negatively correlated with circulating levels of HGF and IL-6. After multivariate regression Log_e_ converted IL-6 was the only factor that was negatively associated with BM CD34^+^-cells (P = 0.007).

### Altered expression of CXCR4 and CD26

**Figure 3:**
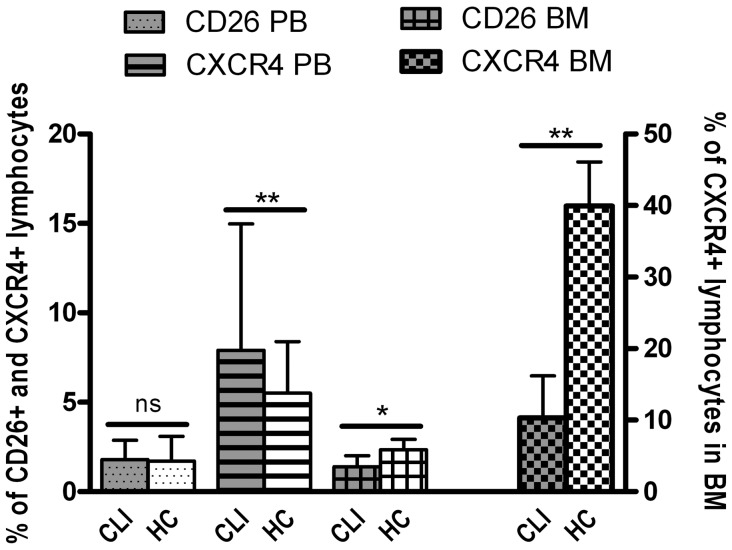
Altered expression of CD26 and CXCR4 in blood and BM of CLI patients. Data represent median and P75. The percentage of CXCR4 expressing lymphocytes was higher in the blood of CLI patients (n = 101) compared to healthy controls (HC; n = 37), while a significant reduction was observed in the BM (n = 101 and n = 12 for CLI patients and healthy controls, respectively). The percentage of CD26 expressing lymphocytes was not different in the PB, while a lower percentage of lymphocytes in the BM expressed CD26 in CLI patients. * P<0.05, ** P<0.01.

Expression of CXCR4 and CD26 on blood and BM cells is involved in the mobilization of progenitor cells from the BM, via the modulation of the response to SDF-1α [25], [26], and was therefore studied in both blood and BM. The percentage of CXCR4 expressing cells in the blood was not different in CLI patients compared to healthy controls for the complete mononuclear cell population (7.5% [4.7–13.4] vs 7.0% [4.6–9.6], P = 0.215), as was the case for mean CXCR4 expression, percentage of CD26 expressing MNC and mean CD26 expression by MNC. The percentage of lymphocytes expressing CXCR4 was significantly higher in CLI patients compared to healthy controls (7.9% [5.2–15.0] vs 5.5% [3.9–8.4], P = 0.004), whereas the percentage of CD26 expressing lymphocytes did not significantly differ (1.8% [1.0–2.9] vs 1.7% [1.2–3.1], P = 0.582) (Fig. 3). The mean CXCR4 expression per monocyte was significantly higher in healthy controls (1.2 [0.9–1.8] vs 1.9 [1.5–2.2], P<0.001), while the percentage of CXCR4 expressing monocytes did not differ significantly. The percentage of CXCR4 and CD26 expressing MNC and lymphocytes in the circulation was not influenced by severity of CLI or presence of diabetes.

In the BM of CLI patients the percentage of MNC expressing CXCR4 (11.1% [7.4–16.8] vs 41.1% [18.5–48.6], P<0.001), and MNC expressing CD26 (1.5% [1.0–2.0] vs 2.3% [1.2–2.7], P = 0.024) and the mean expression of CXCR4 (4.6 [4.4–5.0] vs 8.6 [6.4–12.2], P<0.001) were all lower as compared to controls. The percentage of CXCR4 expressing lymphocytes was significantly lower compared to healthy controls (10.3% [7.3–16.2] vs 40.0% [22.0–46.1], P<0.001), which was also the case for the percentage of CD26 expressing lymphocytes (1.4% [1.0–2.0] vs 2.4% [1.2–2.9], P = 0.026) (Figure 3). In addition mean CXCR4 expression per lymphocyte in the BM was lower in CLI patients (4.6 [4.3–5.1] vs 8.5 [5.8–12.5], P<0.001). No differences were observed for monocytic expression of both CXCR4 and CD26 in the BM. The percentage of CXCR4 expressing MNC and lymphocytes in the BM were both negatively associated with disease severity (rho = −0.255, P = 0.015 and rho = −0.245, P = 0.020), but not with diabetes. CD34^+^KDR^+^ EPC levels both in the circulation as well as in the BM were positively correlated with mean MNC expression of CXCR4 in the BM (rho = 0.323, P = 0.002; rho = 0.370, P<0.001, respectively). This pattern with respect to CD34^+^KDR^+^ EPC levels was also observed for the mean lymphocytic CXCR4 expression in the BM (rho = 0.315, P = 0.003; rho = 0.374, P<0.001, respectively).

The circulating levels of SDF-1α were neither related to levels of CD26 expressing cells in the circulation nor in the BM compartment. Moreover, SDF-1α levels were not related to the percentage of cells expressing CXCR4 in the BM or the circulation.

### Reduced paracrine function despite equal CAC numbers

CAC numbers obtained from cultured PB-MNC were not different between CLI patients and healthy controls (6±8 CAC/1000 MNC vs 10±9 CAC/1000 MNC; P = 0.137). In CLI patients the number of CAC was not affected by the presence of diabetes (5±8 CAC/1000 MNC vs 8±9 CAC/1000 MNC; P = 0.088) and showed no change with disease severity (9±9 CAC/1000 MNC vs 6±6 CAC/1000 MNC; P = 0.116). CAC number was not related to any of the circulating progenitor cell populations.

**Figure 4:**
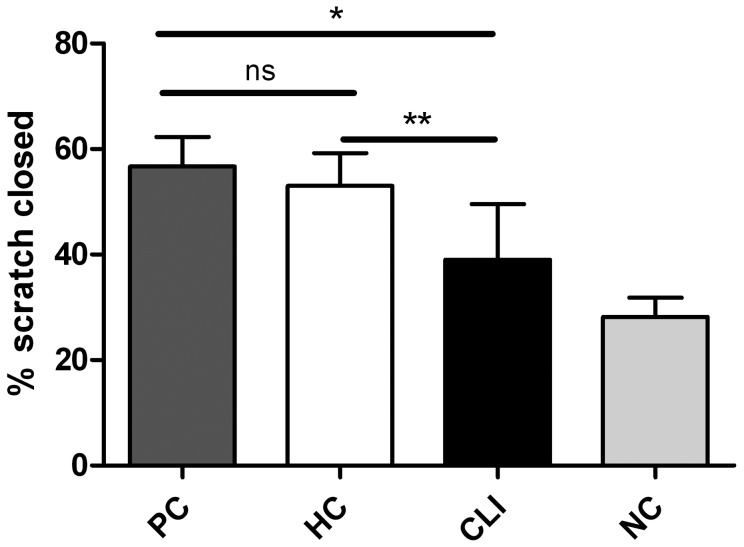
Conditioned medium obtained from CAC cultured from CLI patients has impaired paracrine effects. Data represent median and P75. Percentage of scratch wound closure stimulated with conditioned medium (CM) obtained from CLI derived CAC (n = 33) was significantly reduced (P<0.0001) compared to CM obtained from healthy control (HC; n = 25) derived CAC. Wound closure after stimulation with HC derived CM was equal to stimulation with a positive control (PC; n = 4). CAC serum free culture medium served as a negative control (NC; n = 4). * P<0.05, ** P<0.01.

An important function of CAC during the neovascularization process is the secretion of pro-angiogenic factors [35]. CM obtained from CAC cultures of CLI patients had a significantly reduced capacity to stimulate endothelial cell migration in a scratch wound assay compared to CM obtained from healthy controls (39.7% [28.0–49.8] vs 53.0% [50.0–58.5]; P<0.0001) (Fig. 4). In CLI patients the capability of diabetic CM to stimulate scratch wound closure showed a clear trend to be lower than that of non-diabetic CM (34.3% [11.7–41.3] vs 46.1% [35.1–51.9]; P = 0.052) no differences were observed between patients with Fontaine III or IV (P = 0.882). After correction for CAC number present in the primary culture the presence of CLI remained a significant negative predictor for scratch wound closure (P<0.0001).

## Discussion

Our study shows that circulating EPC levels are significantly reduced in patients with CLI on regular medication, despite significant endothelial injury and upregulation of progenitor cell mobilizing factors in the circulation such as SDF-1α and VEGF. The observed reduction of CD34^+^-cells in the BM of CLI patients suggests that the low circulating EPC levels are at least in part due to BM exhaustion, which appears related to increased systemic inflammation. In addition, BM levels and activity of the progenitor cell mobilizing factor MMP-9 are reduced, indicating that an impairment in the progenitor cell mobilizing response is also involved. Moreover, paracrine function of CAC was impaired. The results of this study suggest that reduced levels and function of circulating progenitor cells and concomitant BM exhaustion and dysfunction contribute to the attenuated neovascularization response in CLI patients.

Our observations of significantly elevated levels of progenitor cell mobilizing chemokines, such as VEGF, SDF-1α, and G-CSF suggest that the ischemic signalling response is not critically disturbed in CLI patients, as has been observed in diabetic animal models [36]. Although our study does not allow definitive mechanistic conclusions, the reduction of BM CD34^+^-cells in CLI patients observed in the present study suggests BM exhaustion as a potential mechanism that contributes to reduced progenitor cell availability in the circulation. It has been suggested that prolonged exposure to pro-inflammatory stimuli may lead to exhaustion or suppression of the progenitor cell pool in the BM [37]–[40], leading to attenuated release of EPC into the circulation and a shift towards the release of more immature and dysfunctional EPC. Indeed, the pro-inflammatory state in CLI with high levels of inflammatory cytokines, such as IL-6, IL-8, and IP-10 [41], [42] and the significant negative correlation between IL-6 levels and CD34^+^ progenitor cell numbers in the BM of CLI patients supports a role for inflammatory suppression or exhaustion of the BM in CLI.

In addition to inflammation-induced suppression or exhaustion of the BM progenitor pool, an impaired mobilization response of progenitor cells from the BM may contribute to the reduction in circulating EPC. We explored how mobilization cues in the BM environment are affected in CLI. The process of EPC mobilization from the BM is critically dependent on MMP-9 [6], [43]. In MMP-9 knockout mice VEGF administration or hindlimb ischemia failed to induce progenitor cell mobilization [6], [44]. The NO-pathway is also essential for the mobilization of BM progenitor cells as eNOS knockout mice show defective VEGF-induced EPC mobilization and profoundly reduced MMP-9 activity, thus phenotypically resembling MMP-9 knockout mice [45]. MMP-9 has been identified as a major target for NO, activating MMP-9 by S-nitrosylation [46]. Patients with cardiovascular diseases, such as CLI have decreased NO bioavailability [47]. Indeed we observed reduced levels of L-arginine, the most important natural occurring substrate for the generation of NO, in CLI, which may negatively affect NO-bioavailability [45], [47]. Furthermore, we show that MMP-9 levels and activity are reduced in the BM of CLI patients. Reduced NO availability in CLI may well extend to the BM compartment and thus — in part – explain the lower MMP-9 levels and activity in CLI patients and hence a reduced mobilization response to tissue ischemia.

The SDF-1α/CXCR4 interaction is another important pathway in the mobilization of progenitor cells from the BM. SDF-1α is a critical mediator for ischemia-specific recruitment of progenitor cells. Gradients of SDF-1α are sensed by cells expressing the SDF-1α receptor CXCR4. We observed altered expression patterns of CXCR4 and CD26 – which is known to inhibit the SDF-1α/CXCR4 interaction [25] – in CLI patients. Since the physical interaction of CXCR4 and SDF-1α retains progenitor cells in their BM niche, disruption of this interaction by CD26 seems an important step in the mobilization of these cells [26]. Therefore the reduced CD26 expression in the BM of CLI patients could hamper the physical release of these cells from the BM to the circulation and thus contribute to a disturbed BM response to tissue ischemia.

In our study, absolute number of CD34^+^KDR^+^ EPC in blood and BM are relatively low, which raises the question about whether these cells could play an important role in neovascularization. However, numbers of CD34^+^KDR^+^ EPC in our control population are similar to reports by others. In our population – if converted to EPC numbers per 10^6^ flow cytometric events – an average of 80 CD34^+^KDR^+^ EPC/10^6^ events was observed, whereas others report 35 to 70 CD34^+^KDR^+^ EPC/10^6^ events [12]; 40.9 to 87.4 CD34^+^KDR^+^ EPC/10^6^ events [13]; 39 to 74 CD34^+^KDR^+^ EPC/10^6^ events [48]. Furthermore, several studies have shown that the number of CD34^+^KDR^+^ EPC correlates with cardiovascular prognosis and disease severity [10]–[13], suggesting a role for these cells in cardiovascular health and vascular regeneration despite the low numbers present in the circulation. Whether this vasculoprotective function is mediated through actions as actual building blocks in neovascularization, or via paracrine pathways [7], [8] cannot be determined by our study.

Other circulating cell types, such as monocytes and lymphocytes, may also be involved in neovascularization [9], [49], [50]. Here we focused on CAC, also known as early EPC, or monocytic EPC. CAC lack characteristics that are required for cells to be considered as true ‘progenitors’, such as a capacity for clonal expansion, and their capability to form sustainable endothelium in vivo has been challenged. However, CAC can be obtained from PB in relatively high numbers and are potent secretors of proangiogenic factors. In contrast to reduced numbers of circulating CD34^+^KDR^+^ EPC, the number of CAC, was not different between CLI patients and healthy controls. CD34^+^KDR^+^ EPC represent a defined subset of true BM-derived progenitor cells with the ability for clonal expansion from single cells into endothelial-like cell colonies [51], [52], whereas CAC are mostly derived from monocytes/macrophages [35]. The pro-inflammatory state in CLI may explain a relatively undisturbed quantity of EPC with inflammatory cell origin. Importantly, we found a marked reduction in their paracrine actions. Such functional impairment of CAC may contribute to the impaired neovascularization response in CLI. Moreover, this may have negative impact on the therapeutic potential of autologous progenitor cell therapy.

In our CLI patients we found a general lack of association of progenitor cell levels with patient characteristics and cardiovascular risk factors, for instance diabetes, which is in contrast to previous reports. This may be explained by the end-stage phase of vascular disease in CLI, where the conditions and risk factors that originally caused and enhanced the development of the disease become overwhelmed by the systemic influence of CLI per se, hence the influence of patient characteristics and cardiovascular risk factors on EPC levels becomes less evident.

This study has limitations, which are in large part related to the limited availability of human BM. We were not able to obtain blood samples from the same control population as where we obtained the BM, ie patients undergoing orthopedic surgery. Therefore our data do not allow conclusions on influence of circulating factors on the BM compartment and vice versa in healthy controls. Furthermore, differences in BM puncture sites between patients and controls, the iliac crest and femoral head respectively, could have influenced composition of the BM. However, we normalized the BM progenitor cell numbers to granulocyte numbers to correct for potential differences in BM composition. No differences were observed in granulocyte numbers between patients and controls.

In conclusion we show that CLI patients, despite profound endothelial injury and an upregulation of progenitor cell mobilizing growth factors and cytokines, have significantly reduced circulating CD34^+^KDR^+^ EPC. Our data suggest that inflammation-induced suppression or exhaustion of the BM progenitor cell pool, as well as a defective progenitor cell mobilization response due to reduced levels and activity of MMP-9 in the BM and alterations in the SDF-1α/CXCR4 interaction contribute to the defective neovascularization response in CLI.

## Supporting Information

Figure S1 **Representative picture of gelatine zymography of BM plasma.** Representative picture of a gelatine zymogram showing increased MMP-9 activity in healthy control (HC) BM plasma compared to BM plasma obtained from CLI patients even on gross inspection. For analysis the lytic zones were normalized to the highest concentration of 83kDA active MMP-9 used as a positive control and expressed as arbitrary units (AU).
(TIF)

Figure S2 **Gating Strategy for detection of CD34^+^ progenitor cells and CD34^+^KDR^+^ EPC.** CD34^+^ progenitor cells were identified in the lymphocytic range of the sideward scatter plot and the KDR^+^-cells in the CD34^+^ gate were defined as CD34^+^KDR^+^ EPC.
(TIF)

Figure S3 **Representative image showing ac-LDL/UEA-lectin double-staining CAC obtained after defined 7-day culture protocol.** PB-MNC after 7-day culture on fibronectin-coated plates in a defined endothelium specific medium acquire endothelial like characteristics and double-staining for ac-LDL/UEA-lectin, specific for CAC. Red = DiI-labeled ac-LDL, Green = FITC-labeled UEA-lectin, Blue = DAPI, Yellow/Orange = double-stain ac-LDL/UEA-lectin.
(TIF)

Table S1 **Univariate correlation of cardiovascular risk factors and endothelial markers and arginine analogues in CLI patients.**
(DOCX)

Table S2 **Univariate correlation of cardiovascular risk factors and chemokines and growth factors in CLI patients.**
(DOCX)

Table S3 **Univariate correlation of cardiovascular risk factors and progenitor cell numbers in CLI patients.**
(DOCX)

Table S4 **Univariate correlation of cardiovascular risk factors and MMP-2 and 9 levels and activity in bone marrow of CLI patients.**
(DOCX)

Appendix S1 **The Juventas Study Group.**
(DOC)
