# Supplementary figures and images for: Bone Marrow Alterations and Lower Endothelial Progenitor Cell Numbers in Critical Limb Ischemia Patients
Source: PLoS One. 2013 Jan 31;8(1):e55592. doi: 10.1371/journal.pone.0055592 (PMC3561321; doi:10.1371/journal.pone.0055592)

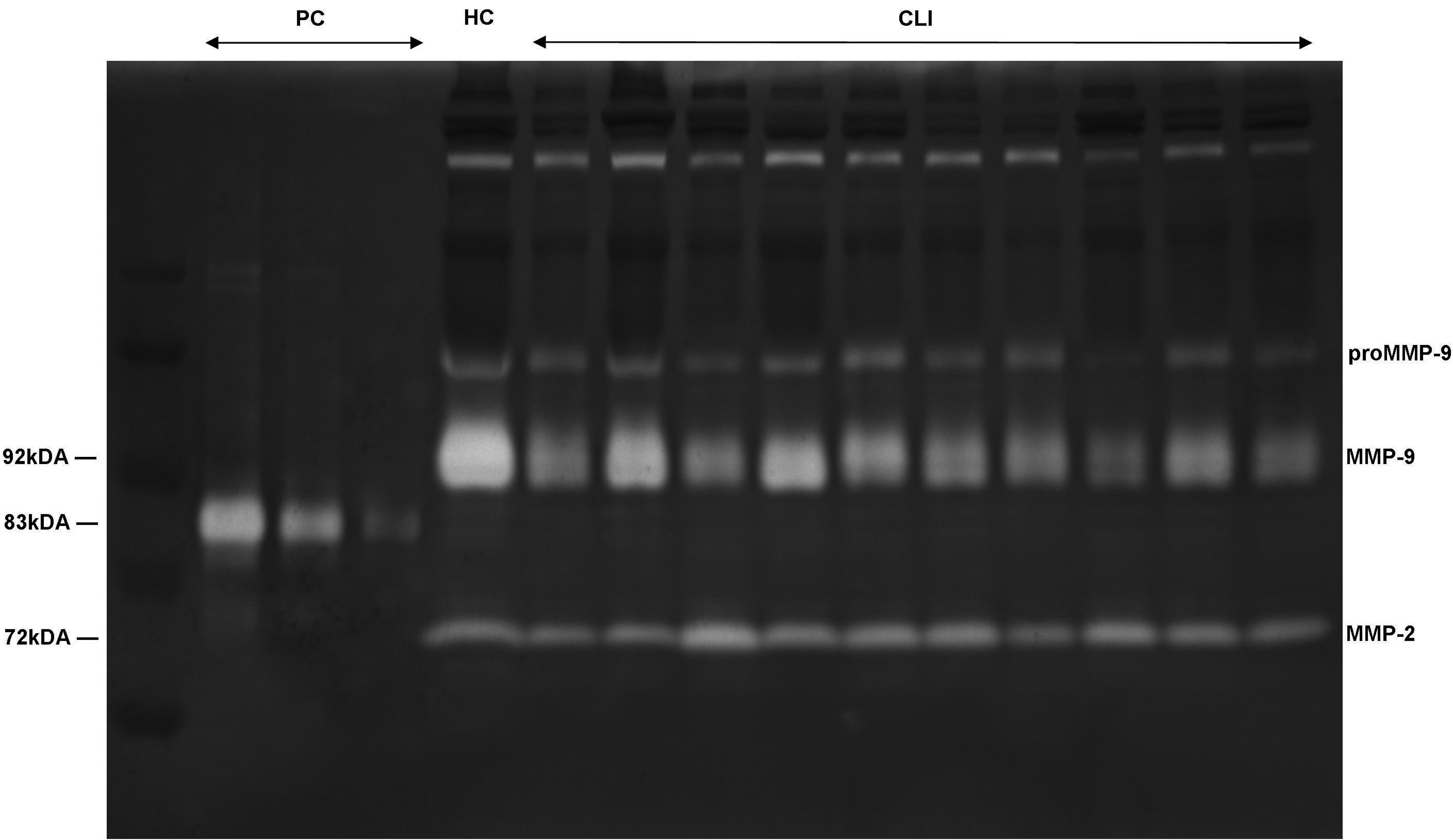

Supplement: Figure S1 — Representative picture of gelatine zymography of BM plasma. Representative picture of a gelatine zymogram showing increased MMP-9 activity in healthy control (HC) BM plasma compared to BM plasma obtained from CLI patients even on gross inspection. For analysis the lytic zones were normalized to the highest concentration of 83kDA active MMP-9 used as a positive control and expressed as arbitrary units (AU). (TIF) [file pone.0055592.s001.tif]

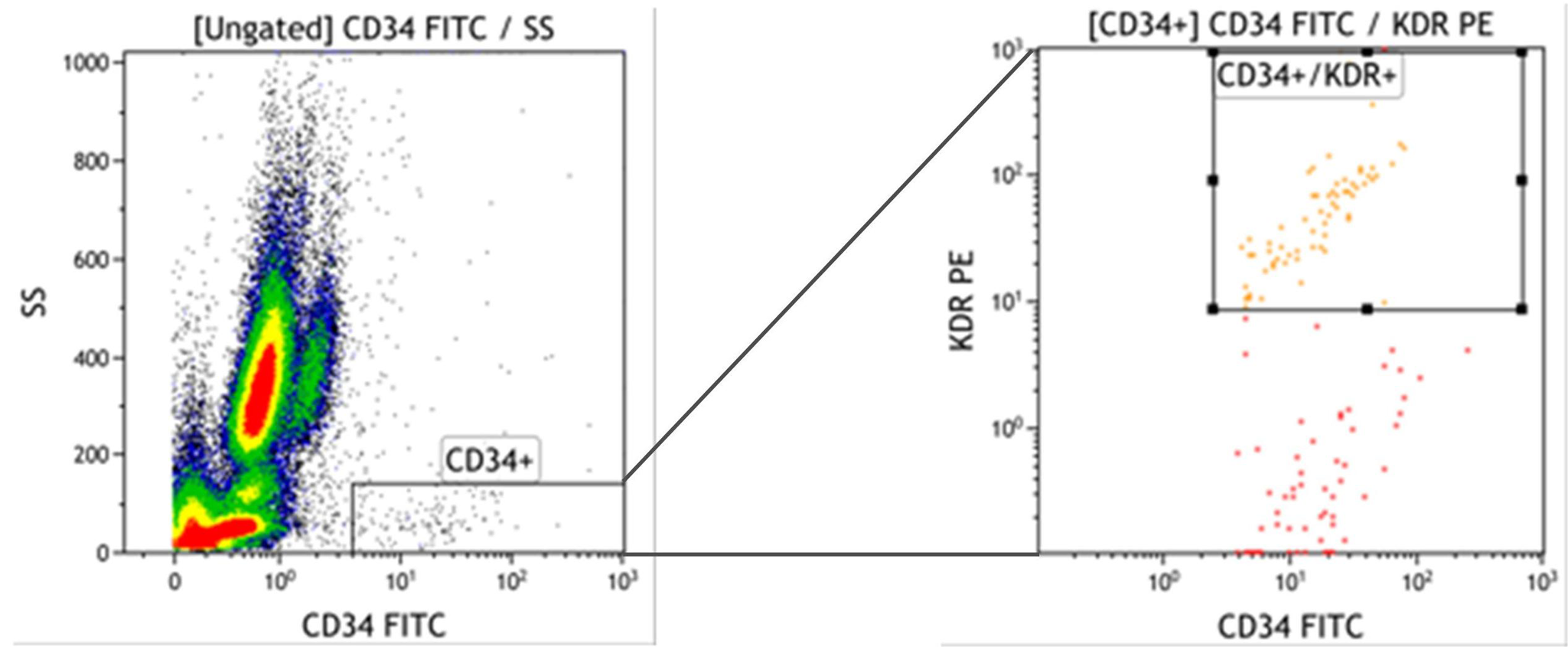

Supplement: Figure S2 — Gating Strategy for detection of CD34+ progenitor cells and CD34+KDR+ EPC. CD34+ progenitor cells were identified in the lymphocytic range of the sideward scatter plot and the KDR+-cells in the CD34+ gate were defined as CD34+KDR+ EPC. (TIF) [file pone.0055592.s002.tif]

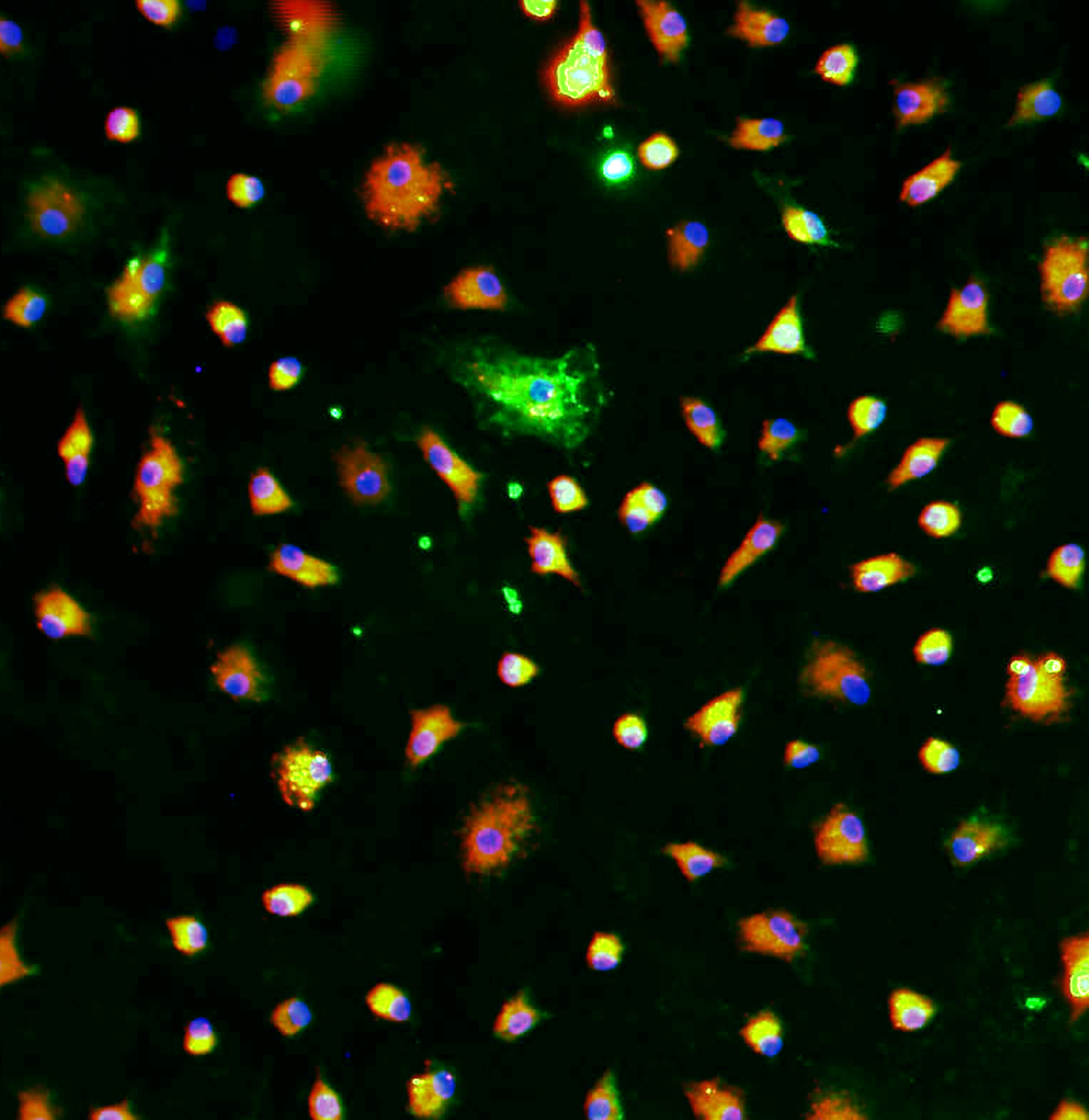

Supplement: Figure S3 — Representative image showing ac-LDL/UEA-lectin double-staining CAC obtained after defined 7-day culture protocol. PB-MNC after 7-day culture on fibronectin-coated plates in a defined endothelium specific medium acquire endothelial like characteristics and double-staining for ac-LDL/UEA-lectin, specific for CAC. Red = DiI-labeled ac-LDL, Green = FITC-labeled UEA-lectin, Blue = DAPI, Yellow/Orange = double-stain ac-LDL/UEA-lectin. (TIF) [file pone.0055592.s003.tif]
