# Supplementary material for: Bone Marrow Alterations and Lower Endothelial Progenitor Cell Numbers in Critical Limb Ischemia Patients
Source: PLoS One. 2013 Jan 31;8(1):e55592. doi: 10.1371/journal.pone.0055592 (PMC3561321; doi:10.1371/journal.pone.0055592)
Supplement: Table S1 — Univariate correlation of cardiovascular risk factors and endothelial markers and arginine analogues in CLI patients. (DOCX) [file pone.0055592.s004.docx]

**Table S1. Univariate correlation of cardiovascular risk factors and endothelial markers and arginine analogues in CLI patients.**

|  | **sE-selectin** | **sICAM-1** | **sVCAM-1** | **thrombomodulin** | **Arginine** | **ADMA** | **SDMA** |
| --- | --- | --- | --- | --- | --- | --- | --- |
| Age | -.21 | -.31* | .27 | .09 | -.09 | .17 | -58** |
| Male gender | -.03 | .17 | -.18 | -.10 | -.08 | -.16 | .04 |
| Body mass index | .07 | .06 | .01 | .09 | -.13 | .14 | -.10 |
| Currently smoking | .09 | .28** | -.20 | -.16 | .03 | -.09 | -.30** |
| Diabetes | .06 | -.16 | .37** | .09 | -.31** | -.01 | .41** |
| Hypertension | .14 | .07 | .08 | .00 | .02 | .03 | .12 |
| Systolic blood pressure | -.06 | -.13 | .07 | .04 | .02 | -.06 | .11 |
| Hypercholesterolemia | -.01 | -.02 | .05 | -.06 | -.04 | .06 | -.03 |
| Total cholesterol | .14 | .16 | -.09 | .06 | .09 | -.03 | -.10 |
| HDL-cholesterol | .14 | .18 | .09 | .03 | .05 | -.21 | .00 |
| LDL-cholesterol | .01 | .10 | -.06 | .14 | .19 | .09 | -.13 |
| Triglycerides | -.11 | -.03 | -.16 | -.12 | -.16 | .03 | -.02 |
| Homocysteine | -.02 | -.16 | .41** | .33* | -.12 | .39** | .66** |
| Creatinine | -.07 | -.14 | .44** | .37** | -.06 | .11 | .66** |
| Fontaine classification (grade III/IV) | .02 | -.02 | .39** | .23 | -.33** | .13 | .26* |
| **Medication use** |  |  |  |  |  |  |  |
| Statins | .16 | .14 | -.25 | .14 | .16 | -.24* | -.14 |
| ACEI/ARB | .20 | -.17 | .17 | .21 | .07 | .20 | .35** |
| Beta-blockers | -.16 | -.02 | -.06 | -.08 | -.13 | .07 | .09 |
| Diuretics | .15 | -.03 | .16 | .36* | -.11 | .11 | .33** |
| Anticoagulants | -.09 | -.25 | .07 | -.29* | -.30** | .02 | -.12 |
| APT | -.01 | .15 | -.19 | .10 | .27* | -.01 | .01 |

Data represent Spearman’s rho or point-biserial correlation coefficients (r_pb_) in case one of the variables is nominal. Presence of hypertension, hypercholesterolemia, and hyperhomocysteinemia were determined at the time of inclusion. Hypertension was defined as having a systolic blood pressure >140 mmHg or taking antihypertensive medication. Hypercholesterolemia was defined as having a total cholesterol level >6.5 mmol/l or taking cholesterol reducing medication. ACEI/ARB=ACE inhibitor or angiotensin receptor blocker. APT=Antiplatelet therapy. Green cells indicate significant positive correlations and red cells significant negative correlations. * P<0.05, ** P<0.01
