# Supplementary material for: Bone Marrow Alterations and Lower Endothelial Progenitor Cell Numbers in Critical Limb Ischemia Patients
Source: PLoS One. 2013 Jan 31;8(1):e55592. doi: 10.1371/journal.pone.0055592 (PMC3561321; doi:10.1371/journal.pone.0055592)
Supplement: Table S2 — Univariate correlation of cardiovascular risk factors and chemokines and growth factors in CLI patients. (DOCX) [file pone.0055592.s005.docx]

**Table S2. Univariate correlation of cardiovascular risk factors and chemokines and growth factors in CLI patients.**

|  | **FGF-b** | **G-CSF** | **GRO-a** | **HGF** | **IL-6** | **IL-8** | **IP-10** | **MCP-1** | **PDGF-bb** | **RANTES** | **SCF** | **SCGF-β** | **SDF-1α** | **TNF-α** | **TRAIL** | **VEGF-A** |
| --- | --- | --- | --- | --- | --- | --- | --- | --- | --- | --- | --- | --- | --- | --- | --- | --- |
| Age | -.21* | -.08 | -.01 | -.01 | .02 | -.01 | .17 | -.17 | -.16 | -.24* | .20* | -.14 | .21* | -.09 | -.03 | -.10 |
| Male gender | .07 | -.12 | .06 | .00 | -.08 | .02 | .12 | -.10 | -.06 | .00 | -.15 | -.12 | -.06 | -.05 | .08 | -.18 |
| Body mass index | -.06 | .04 | .08 | .14 | -.04 | -.02 | .02 | .00 | .02 | .15 | .03 | -.14 | .08 | -.09 | .13 | .12 |
| Currently smoking | .04 | .05 | -.05 | .03 | -.06 | -.16 | -.37** | .05 | .15 | .21* | -.19 | .11 | -.10 | -.12 | -.06 | .08 |
| Diabetes | -.06 | -.08 | .19 | .19 | .21* | .20* | .10 | -.06 | -.24* | -.08 | .34** | -.11 | .16 | .08 | -.02 | .08 |
| Hypertension | -.06 | -.09 | .01 | .06 | .02 | -.01 | .11 | -.02 | -.11 | -.16 | .12 | .10 | .01 | -.12 | .03 | -.02 |
| Systolic blood pressure | -.13 | -.14 | -.20* | -.12 | -.26** | -.17 | .02 | .04 | -.11 | .24* | -.05 | .18 | -.03 | -.20* | .13 | -.17 |
| Hypercholesterolemia | -.11 | .00 | -.09 | .04 | -.10 | -.09 | -.12 | -.07 | .04 | .08 | .12 | -.06 | .00 | -.17 | .20* | -.09 |
| Total cholesterol | -.20* | -.30** | -.29** | -.23* | -.32** | -.27** | -.03 | -.17 | -.08 | .06 | -.18 | -.08 | -.34** | -.21* | .21* | -.33** |
| HDL-cholesterol | -.07 | -.11 | -.24* | -.26* | -.24* | -.23* | .01 | -.08 | .08 | .01 | -.31** | -.10 | -.22* | -.13 | -.01 | -.24* |
| LDL-cholesterol | -.11 | -.21* | -.27** | -.27** | -.17 | -.25* | -.13 | -.09 | .01 | .04 | -.19 | .01 | -.32** | -.10 | .19 | -.24* |
| Triglycerides | -.13 | -.13 | .05 | .14 | -.11 | .02 | .10 | -.06 | -.17 | -.02 | .15 | .02 | .01 | -.10 | .23* | .05 |
| Homocysteine | -.11 | -.12 | .14 | .21* | .12 | .21* | .16 | -.10 | -.06 | -.19 | .36** | .03 | .32** | .04 | -.04 | -.02 |
| Creatinine | -.19 | -.03 | .01 | .05 | .04 | .02 | .10 | -.01 | -.11 | -.15 | .67** | .12 | .46** | -.07 | -.12 | -.01 |
| Fontaine classification | .02 | -.06 | .20* | .11 | .30** | .19 | -.10 | -.03 | -.16 | -.34** | .22* | -.14 | .12 | .11 | -.08 | .10 |
| **Medication use** |  |  |  |  |  |  |  |  |  |  |  |  |  |  |  |  |
| Statins | -.02 | .06 | .06 | -.10 | -.03 | -.02 | -.03 | .13 | -.11 | -.13 | .04 | -.11 | -.02 | -.09 | .00 | -.07 |
| ACEI/ARB | -.21* | -.16 | .00 | -.11 | -.06 | -.16 | -.04 | -.02 | -.33** | -.11 | .19 | -.01 | .13 | -.12 | -.09 | -.11 |
| Beta-blockers | -.19 | -.09 | .06 | -.04 | -.07 | -.04 | .09 | -.01 | -.27** | -.14 | .17 | -.14 | .15 | -.20* | -.08 | -.03 |
| Diuretics | .02 | -.07 | -.07 | .13 | -.01 | .03 | .10 | -.19 | -.10 | -.01 | .31** | .09 | .18 | .02 | -.04 | -.07 |
| Anticoagulants | .16 | .01 | .07 | .14 | .12 | .21* | .18 | -.10 | -.17 | .14 | .10 | .00 | .06 | -.01 | -.02 | -.05 |
| APT | -.04 | .13 | -.05 | -.12 | -.02 | -.05 | -.11 | .23* | .16 | -.03 | -.15 | -.06 | -.07 | .11 | -.10 | .09 |

Data represent Spearman’s rho or point-biserial correlation coefficients (r_pb_) in case one of the variables is nominal. Presence of hypertension, hypercholesterolemia, and hyperhomocysteinemia were determined at the time of inclusion. Hypertension was defined as having a systolic blood pressure >140 mmHg or taking antihypertensive medication. Hypercholesterolemia was defined as having a total cholesterol level >6.5 mmol/l or taking cholesterol reducing medication. ACEI/ARB=ACE inhibitor or angiotensin receptor blocker. APT=Antiplatelet therapy. Green cells indicate significant positive correlations and red cells significant negative correlations. * P<0.05, ** P<0.01
