# Supplementary material for: Bone Marrow Alterations and Lower Endothelial Progenitor Cell Numbers in Critical Limb Ischemia Patients
Source: PLoS One. 2013 Jan 31;8(1):e55592. doi: 10.1371/journal.pone.0055592 (PMC3561321; doi:10.1371/journal.pone.0055592)
Supplement: Table S3 — Univariate correlation of cardiovascular risk factors and progenitor cell numbers in CLI patients. (DOCX) [file pone.0055592.s006.docx]

**Table S3. Univariate correlation of cardiovascular risk factors and progenitor cell numbers in CLI patients.**

|  | **Peripheral Blood** | | | | **Bone Marrow** | | | | **CAC** | |
| --- | --- | --- | --- | --- | --- | --- | --- | --- | --- | --- |
|  | **CD34** | **%KDR of CD34** | **CD34KDR** | **CD133** | **CD34** | **%KDR of CD34** | **CD34KDR** | **CD133** | **CAC** | **Paracrine effect (n=32)** |
| Age | -.29** | .10 | -.07 | -.26** | -.13 | .03 | .00 | -.11 | -.12 | -.04 |
| Male gender | -.08 | .07 | -.01 | .15 | .01 | .12 | .13 | .03 | -.06 | -.02 |
| Body mass index | -.07 | -.03 | -.08 | -.06 | -.01 | -.03 | -.06 | -.10 | .13 | -.21 |
| Currently smoking | .00 | .11 | .16 | .14 | -.02 | .08 | .08 | -.13 | .06 | .02 |
| Diabetes | -.19 | -.03 | -.14 | -.34** | -.15 | .18 | .10 | -.07 | -.18 | -.36* |
| Hypertension | .11 | .02 | .14 | .05 | -.05 | -.04 | -.07 | -.05 | .00 | -.07 |
| Systolic blood pressure | .11 | .08 | .20* | .07 | -.04 | -.03 | -.07 | -.09 | .07 | .03 |
| Hypercholesterolemia | -.11 | .21* | .15 | .07 | -.14 | .15 | .10 | -.14 | -.14 | N/A |
| Total cholesterol | .04 | .00 | .04 | .06 | .20 | -.16 | -.06 | .07 | .07 | -.31 |
| HDL-cholesterol | .01 | -.03 | -.01 | .01 | .10 | .02 | .10 | .17 | .05 | .27 |
| LDL-cholesterol | .05 | -.03 | .04 | .18 | .11 | -.21* | -.15 | .07 | .02 | -.33 |
| Triglycerides | .13 | .03 | .05 | -.04 | .20* | -.11 | -.03 | -.05 | .09 | -.31 |
| Homocysteine | -.13 | .10 | .04 | -.05 | -16 | .00 | -.08 | -.12 | -.12 | -.20 |
| Creatinine | -.05 | .03 | .02 | -.15 | -.18 | .02 | -.03 | -.26* | -.14 | -.05 |
| Fontaine classification (grade III/IV) | -.08 | -.11 | -.10 | -.26** | -.20* | -.04 | -.13 | -.07 | -.16 | .03 |
| **Medication use** |  |  |  |  |  |  |  |  |  |  |
| Statins | .04 | -.13 | -.10 | -.17 | -.08 | -.05 | -.14 | -.02 | .14 | .39* |
| ACEI/ARB | .07 | -.06 | -.04 | -.03 | .00 | .02 | .03 | .03 | -.10 | -.16 |
| Beta-blockers | .13 | -.03 | .04 | .05 | .09 | -.19 | -.16 | -.02 | .08 | .06 |
| Diuretics | -.07 | .01 | -.07 | -.11 | -.14 | .03 | -.01 | .01 | -.18 | -.47** |
| Anticoagulants | .10 | .13 | .21* | .14 | -.09 | .20* | .14 | .01 | .08 | .09 |
| APT | -.12 | -.05 | -.17 | -.13 | .04 | -.14 | -.13 | -.10 | .03 | -.05 |

Data represent Spearman’s rho or point-biserial correlation coefficients (r_pb_) in case one of the variables is nominal. Presence of hypertension, hypercholesterolemia, and hyperhomocysteinemia were determined at the time of inclusion. Hypertension was defined as having a systolic blood pressure >140 mmHg or taking antihypertensive medication. Hypercholesterolemia was defined as having a total cholesterol level >6.5 mmol/l or taking cholesterol reducing medication. ACEI/ARB=ACE inhibitor or angiotensin receptor blocker. APT=Antiplatelet therapy. Green cells indicate significant positive correlations and red cells significant negative correlations. * P<0.05, ** P<0.01
