# Supplementary material for: Bone Marrow Alterations and Lower Endothelial Progenitor Cell Numbers in Critical Limb Ischemia Patients
Source: PLoS One. 2013 Jan 31;8(1):e55592. doi: 10.1371/journal.pone.0055592 (PMC3561321; doi:10.1371/journal.pone.0055592)
Supplement: Table S4 — Univariate correlation of cardiovascular risk factors and MMP-2 and 9 levels and activity in bone marrow of CLI patients. (DOCX) [file pone.0055592.s007.docx]

**Table S4. Univariate correlation of cardiovascular risk factors and MMP-2 and 9 levels and activity in bone marrow of CLI patients.**

|  | **Levels** | | **Activity** | | |
| --- | --- | --- | --- | --- | --- |
|  | **MMP-2** | **MMP-9** | **MMP-2** | **Pro-MMP-9** | **MMP-9** |
| Age | .33** | -.09 | -.02 | .02 | -.07 |
| Male gender | -.09 | .12 | -.14 | -.07 | -.10 |
| Body mass index | -.03 | .03 | -.22 | -.14 | -.07 |
| Currently smoking | -.34** | .22** | -.02 | .08 | .02 |
| Diabetes | .45** | -.08 | .21 | -.02 | -.15 |
| Hypertension | -.10 | -.11 | .02 | -.18 | -.14 |
| Systolic blood pressure | -.08 | -.04 | .13 | -.04 | -.03 |
| Hypercholesterolemia | -.11 | .18 | .04 | .05 | .02 |
| Total cholesterol | -.26** | -.08 | -.19 | -.10 | .00 |
| HDL-cholesterol | .05 | -.12 | -.03 | -.18 | -.17 |
| LDL-cholesterol | -.30** | -.08 | -.11 | -.08 | .03 |
| Triglycerides | -.13 | .06 | -.12 | .01 | .07 |
| Homocysteine | .31** | .07 | .14 | .20 | .15 |
| Creatinine | .44** | -.02 | .28* | -.15 | -.11 |
| Fontaine classification (grade III/IV) | .33** | -.04 | .22 | .02 | .08 |
| **Medication use** |  |  |  |  |  |
| Statins | -.13 | -.13 | -.19 | -.15 | -.17 |
| ACEI/ARB | .26** | -.13 | .31* | .08 | .08 |
| Beta-blockers | .06 | .00 | .19 | .04 | .13 |
| Diuretics | .15 | .00 | .13 | .03 | .05 |
| Anticoagulants | -.08 | .07 | -.05 | .03 | .19 |
| APT | .06 | -.10 | .03 | .03 | -.06 |

Data represent Spearman’s rho or point-biserial correlation coefficients (r_pb_) in case one of the variables is nominal. Presence of hypertension, hypercholesterolemia, and hyperhomocysteinemia were determined at the time of inclusion. Hypertension was defined as having a systolic blood pressure >140 mmHg or taking antihypertensive medication. Hypercholesterolemia was defined as having a total cholesterol level >6.5 mmol/l or taking cholesterol reducing medication. ACEI/ARB=ACE inhibitor or angiotensin receptor blocker. APT=Antiplatelet therapy. Green cells indicate significant positive correlations and red cells significant negative correlations. * P<0.05, ** P<0.01
