# Supplementary material for: Bone Marrow Alterations and Lower Endothelial Progenitor Cell Numbers in Critical Limb Ischemia Patients
Source: PLoS One. 2013 Jan 31;8(1):e55592. doi: 10.1371/journal.pone.0055592 (PMC3561321; doi:10.1371/journal.pone.0055592)
Supplement: Appendix S1 — The Juventas Study Group. (DOC) [file pone.0055592.s008.doc]

## Appendix I

Members of the JUVENTAS Study Group are:

**M. Teraa, MD**

Department of Vascular Surgery, University Medical Center Utrecht, Utrecht, The Netherlands

Department of Nephrology & Hypertension, University Medical Center Utrecht, Utrecht, The Netherlands

**R.W. Sprengers, MD PhD**

Department of Radiology, University Medical Center Utrecht, Utrecht, The Netherlands

**M.C. Verhaar, MD PhD**

Department of Nephrology & Hypertension, University Medical Center Utrecht, Utrecht, The Netherlands

**F.L. Moll, MD PhD**

Department of Vascular Surgery, University Medical Center Utrecht, Utrecht, The Netherlands

**R.E.G. Schutgens, MD PhD**

Department of Haematology, University Medical Center Utrecht, Utrecht, The Netherlands

**I.C.M. Slaper-Cortenbach, PhD**

Cell Therapy Facility, Department of Pharmacy, University Medical Center Utrecht, Utrecht, The Netherlands

**Y. van der Graaf, MD PhD**

Julius Center for Health Sciences and Primary Care, University Medical Center Utrecht, Utrecht, The Netherlands

**P.A. Doevendans, MD PhD**

Department of Cardiology, University Medical Center Utrecht, Utrecht, The Netherlands

**W.P.Th.M. Mali, MD PhD**

Department of Radiology, University Medical Center Utrecht, Utrecht, The Netherlands
